# Time series radiomics for the prediction of prostate cancer progression in patients on active surveillance

**DOI:** 10.1007/s00330-023-09438-x

**Published:** 2023-02-07

**Authors:** Nikita Sushentsev, Leonardo Rundo, Luis Abrego, Zonglun Li, Tatiana Nazarenko, Anne Y. Warren, Vincent J. Gnanapragasam, Evis Sala, Alexey Zaikin, Tristan Barrett, Oleg Blyuss

**Affiliations:** 1 Department of Radiology, School of Clinical Medicine, Addenbrooke’s Hospital, University of Cambridge, Cambridge Biomedical Campus, Box 218, Cambridge, CB2 0QQ UK; 2 Department of Information and Electrical Engineering and Applied Mathematics (DIEM), University of Salerno, Fisciano, SA Italy; 3 Department of Women’s Cancer, Institute for Women’s Health, University College London, London, UK; 4 Department of Mathematics, University College London, London, UK; 5 Department of Pathology, Cambridge University Hospitals NHS Foundation Trust, Cambridge, UK; 6 Department of Urology, Cambridge University Hospitals NHS Foundation Trust, Cambridge, UK; 7 Cambridge Urology Translational Research and Clinical Trials Office, Addenbrooke’s Hospital, Cambridge Biomedical Campus, Cambridge, UK; 8 Cancer Research UK Cambridge Institute, University of Cambridge, Cambridge, UK; 9 Wolfson Institute of Population Health, Queen Mary University of London, London, UK; 10 Center of Photonics, Lobachevsky University, Nizhny Novgorod, Russian Federation

**Keywords:** Prostatic neoplasms, Magnetic resonance imaging, Artificial intelligence

## Abstract

**Abstract:**

Serial MRI is an essential assessment tool in prostate cancer (PCa) patients enrolled on active surveillance (AS). However, it has only moderate sensitivity for predicting histopathological tumour progression at follow-up, which is in part due to the subjective nature of its clinical reporting and variation among centres and readers. In this study, we used a long short-term memory (LSTM) recurrent neural network (RNN) to develop a time series radiomics (TSR) predictive model that analysed longitudinal changes in tumour-derived radiomic features across 297 scans from 76 AS patients, 28 with histopathological PCa progression and 48 with stable disease. Using leave-one-out cross-validation (LOOCV), we found that an LSTM-based model combining TSR and serial PSA density (AUC 0.86 [95% CI: 0.78–0.94]) significantly outperformed a model combining conventional delta-radiomics and delta-PSA density (0.75 [0.64–0.87]; *p* = 0.048) and achieved comparable performance to expert-performed serial MRI analysis using the Prostate Cancer Radiologic Estimation of Change in Sequential Evaluation (PRECISE) scoring system (0.84 [0.76–0.93]; *p* = 0.710). The proposed TSR framework, therefore, offers a feasible quantitative tool for standardising serial MRI assessment in PCa AS. It also presents a novel methodological approach to serial image analysis that can be used to support clinical decision-making in multiple scenarios, from continuous disease monitoring to treatment response evaluation.

**Key Points:**

•*LSTM RNN can be used to predict the outcome of PCa AS using time series changes in tumour-derived radiomic features and PSA density.*

•*Using all available TSR features and serial PSA density yields a significantly better predictive performance compared to using just two time points within the delta-radiomics framework.*

*•The concept of TSR can be applied to other clinical scenarios involving serial imaging, setting out a new field in AI-driven radiology research.*

**Supplementary Information:**

The online version contains supplementary material available at 10.1007/s00330-023-09438-x.

## Introduction

Prostate cancer (PCa) is the second commonest and the fifth deadliest male cancer globally [1]. Nearly half of newly diagnosed men present with low-risk and favourable intermediate-risk disease [2], for which active surveillance (AS) is the recommended treatment option [3]. However, 27% of AS patients show 5-year histopathological disease progression [4], highlighting the unmet clinical need for improved baseline and follow-up risk stratification. MRI at baseline has been shown to be of benefit in selecting for AS by identifying and excluding patients in whom AS is inappropriate [5, 6]. Simultaneously, the pooled performance of serial MRI for follow-up AS monitoring is only moderate and highly variable [7], in part due to the inherently subjective and reader-dependent nature of its assessment that is particularly evident in non-specialist centres [8]. To address this, the standardised 5-point Prostate Cancer Radiological Estimation of Change in Sequential Evaluation (PRECISE) score [9] was introduced. Despite becoming a gold standard for serial MRI assessment and demonstrating a high negative predictive value ranging between 92 and 100% [10], PRECISE has been exclusively validated in expert centres and still showed only a moderate positive predictive value of 15 to 66% for predicting histopathological PCa progression on AS [10].

In parallel with working on further iterations of PRECISE, developing quantitative tools to make serial MRI assessment more objective may help further improve its performance and limit variance to achieve consistent expert-level quality [6]. Pilot studies have adopted artificial intelligence (AI) techniques to devise MRI-derived radiomics models for predicting PCa progression on AS both at baseline [11] and at follow-up [12]. However, in the multiple time point follow-up setting, the only established methodological framework for analysing temporal radiomic patterns is delta-radiomics (DR), which only measures change between two time points [13]. Being more appropriate for treatment response assessment where clinical decisions are often based on a single post-treatment study, it is less applicable to the AS setting where patients undergo multiple scans, each storing quantitative data describing patient-specific dynamics of tumour development.

In this study, we aimed to develop a time series radiomics (TSR) framework for predicting histopathological PCa progression on AS based on longitudinal changes in radiomic features extracted from all MRI scans obtained over the follow-up period. We hypothesised that the TSR predictive model incorporating all imaging and clinical data collected during AS would outperform DR and achieve at least comparable performance to the expert-derived PRECISE scoring as a clinical standard, offering a novel approach towards quantitative serial medical imaging data analysis.

## Materials and methods

### Dataset and study population

This ethically approved (Health Research Authority and Health and Care Research Wales, IRAS Project ID 288,185) retrospective exploratory study included consecutive patients with biopsy-proven PCa enrolled on AS in our institution between July 2013 and October 2019 according to the previously published criteria [14], which are limited to the inclusion of International Society of Urogenital Pathology (ISUP) grade groups 1 and 2 disease with ≤ 10% Gleason pattern 4. The minimum inclusion criteria for this study were the presence of at least one MR-visible lesion, 2-year follow-up, three 3 T MRI scans (same magnet), and one repeat targeted biopsy within 12 months of the final MRI. The exclusion criteria are described in Supplementary Information. AS progression was defined as biopsy-proven histopathological ISUP grade group progression.

### MRI acquisition

All patients underwent prostate MRI on a 3 T MR750 scanner (GE Healthcare) using a 32-channel receiver coil, with the full protocol described in Supplementary Information. At baseline, all patients underwent multiparametric MRI protocol that included multiplanar high-resolution T_2_-weighted, diffusion-weighted imaging (DWI), and dynamic contrast-enhanced (DCE) MRI. Follow-up scans were acquired using a biparametric MRI protocol, omitting DCE-MRI.

### Ground truth assessment

Since AS eligibility is primarily based on tumour histopathological characteristics, repeat targeted biopsy results were selected as an appropriate ground truth reference standard for confirming the disease progression [15]. Both baseline and follow-up biopsies were targeted at the same lesions delineated on MRI, with biopsy reports including separate sections dedicated to the assessment of target cores. Repeat biopsy procedures were either protocol-driven (12 and 36 months after AS enrolment) or triggered by either three consecutive elevated serum prostate-specific antigen (PSA) levels that were above the pre-defined threshold or radiological progression (PRECISE score ≥ 4) [9]. Biopsy samples were reviewed by an expert genitourinary pathologist (A.Y.W.) according to ISUP guidelines.

### Image segmentation and analysis

**Fig. 1:**
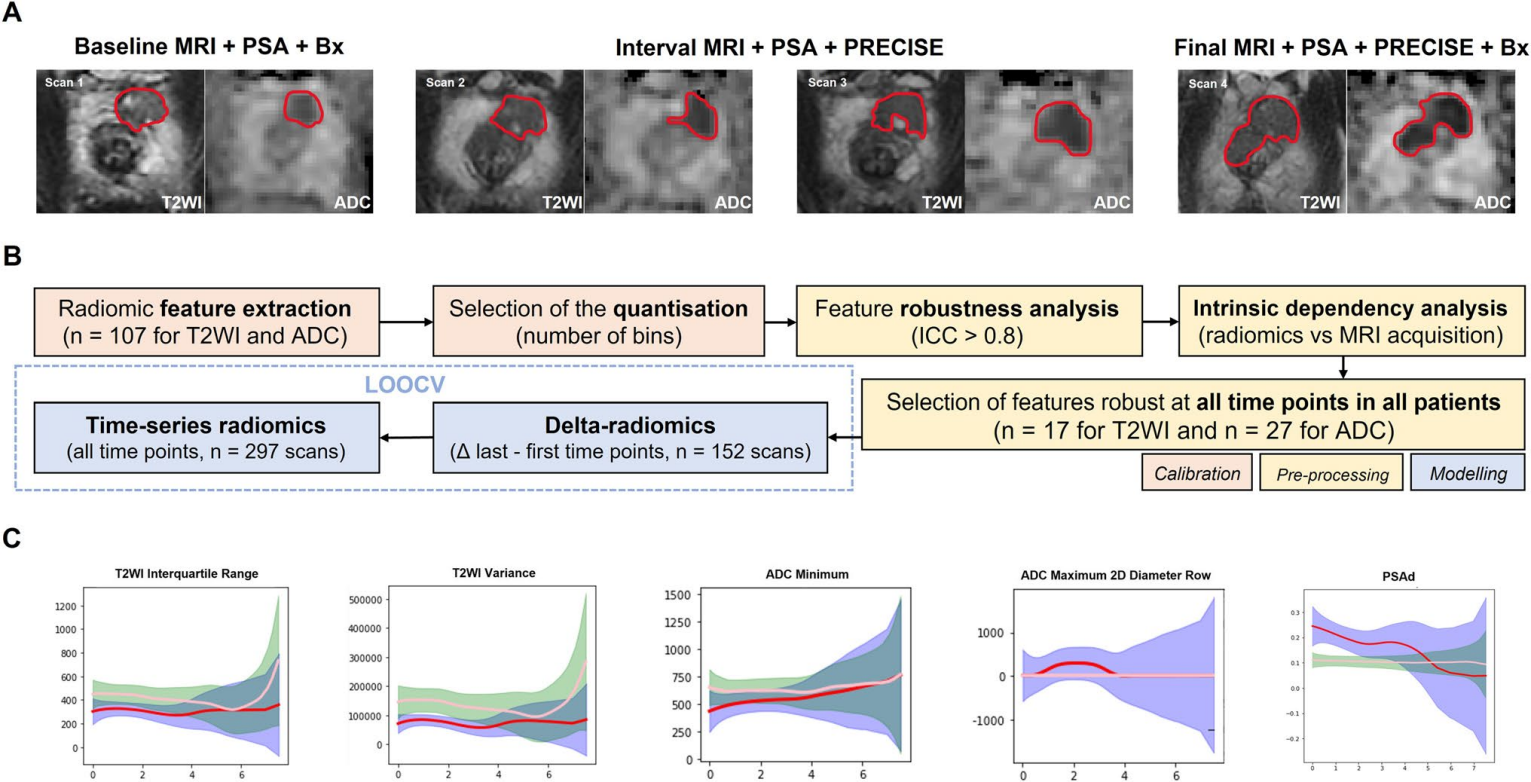
Example T2WI and ADC images (**A**) obtained from a patient with a PZ lesion (red ROI) that showed progression from ISUP grade group 1 at baseline to ISUP grade group 3 at repeat biopsy triggered by radiological progression noted at scan 4. Flow diagram (**B**) summarising the key stages of radiomic analysis used in this study, from radiomic feature extraction to leave-one-out cross-validation (LOOCV) of baseline radiomics, delta-radiomics, and time series radiomics predictive models. Locally weighted scatterplot smoothing (LOWESS) curves (**C**) demonstrating time series changes in representative T2WI- and ADC-derived radiomic features and PSA density in progressors (red lines) and non-progressors (yellow lines), with x-axes denoting years to progression or end of follow-up and y-axes denoting corresponding values

Tumour regions of interest (ROIs) were drawn on de-identified anatomical T2WI and ADC maps (Fig. 1A) by a fellowship-trained urogenital radiologist (T.B.) with a 14-year clinical experience in prostate MRI and a research fellow (N.S.) with a 5-year experience. ROIs were drawn in consensus using open-source segmentation software ITK-SNAP [16], matching the original ROIs used for targeted biopsy of suspicious lesions with a PI-RADS score of ≥ 3.

Follow-up MRI scans were prospectively scored on a 5-point PRECISE scale [9] by four expert urogenital radiologists with 6–17-year experience in prostate MRI reporting and considered experts based on the number of MRIs reported [17, 18]. For predictive modelling, the PRECISE scores were dichotomised at a cut-off value of 4, with patients scoring ≥ 4 at any point deemed as those exhibiting radiological PCa progression. At the time of clinical reporting of the PRECISE scores, the readers were aware of clinical information that included PSA and PSA density dynamics, which reflects the real-life clinical use of this scoring system.

### Image calibration and pre-processing

Figure 1B summarises the radiomics pipeline used to develop and validate DR and TSR predictive models for PCa progression on AS. Tumour-derived radiomic features were extracted using PyRadiomics version 2.0 and Python version 3.7.5 [19]; full features are presented in Supplementary Information. Only features deemed robust at all time points for all patients, separately for T2WI (*n* = 17) and ADC (*n* = 27), were included for predictive modelling (Supplementary Information), with representative examples shown in Fig. 1C.

### Prostate-specific antigen predictive modelling

The addition of non-radiomic clinical parameters to texture features is expected to result in more holistic predictive models [20]. We retrieved all serial prostate-specific antigen (PSA) measurements and derived PSA density (PSAd; Fig. 1C) as a known independent baseline predictor used in clinical practice [14, 15], which was prioritised for predictive modelling following the rationale described in Supplementary Information.

### Delta-radiomics predictive modelling

DR features were computed as the difference between the final and baseline features as:

$$\triangle f=f_{\mathrm{final}}-f_{\mathrm{base}}.$$

where ${f}_{\mathrm{base}}$ and ${f}_{\mathrm{final}}$ correspond to features derived from baseline and final scans, respectively. Predictive modelling was employed using parenclitic networks [21] as described previously [12].

### Time series radiomics predictive modelling

**Fig. 2:**
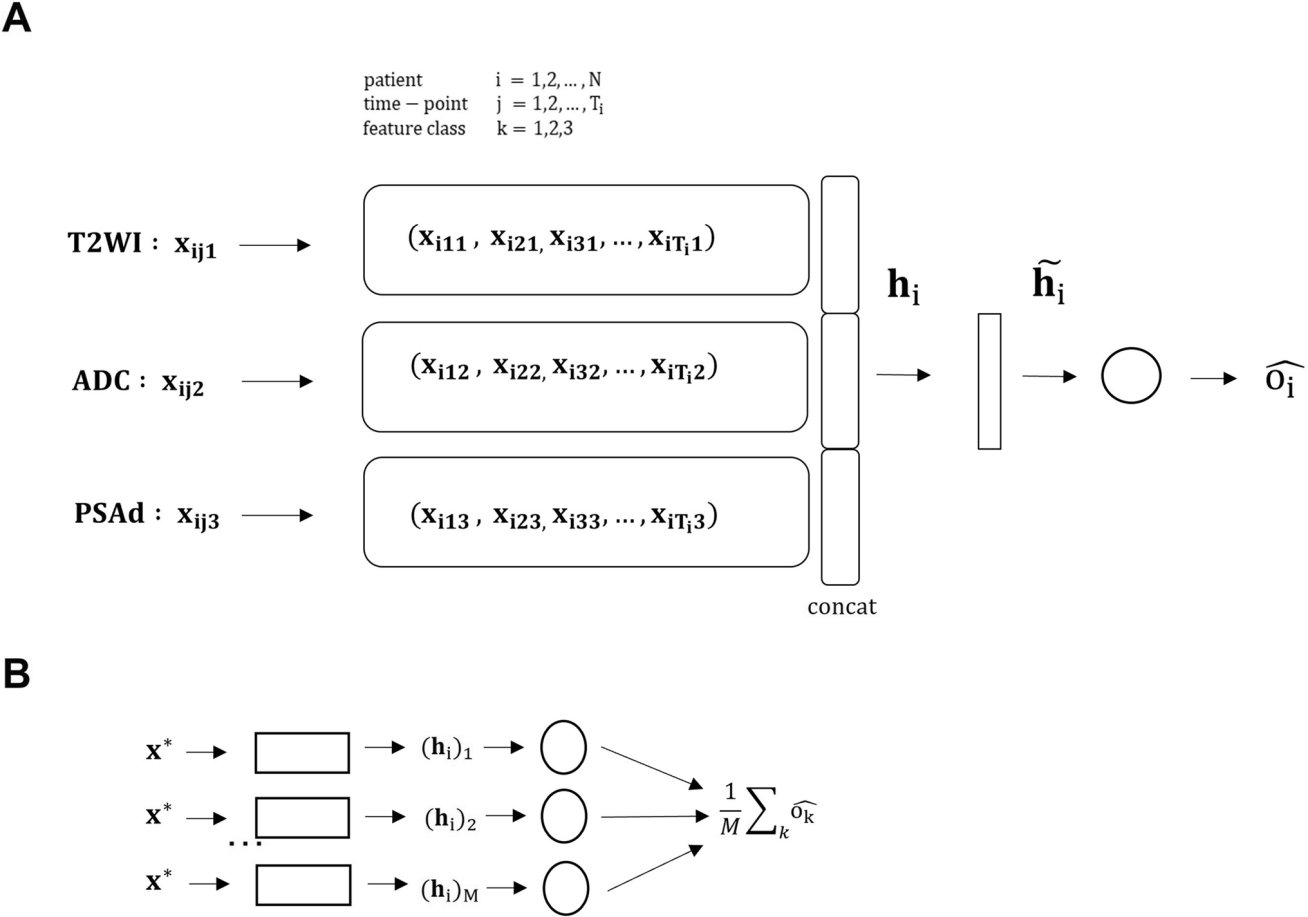
Multistream classification using longitudinal data. The input (**A**) consists of multiple parallel synchronised input sequences ${{\varvec{x}}}_{\mathrm{ijk}}$ grouped into T2WI-derived radiomic features, ADC-derived radiomic features, and PSA density. Features were first standardised and fed into their respective LSTM cell. Then, we concatenated the last hidden state ${{\varvec{h}}}_{\mathrm{i}}$, which was associated with the LSTM output from each clinical feature. We computed $\widetilde{{{\varvec{h}}}_{\mathrm{i}}}$ by adding a dropout layer during training. The final output $\widehat{{o}_{i}}$ was estimated from a regular densely connected neural network layer of 1 unit with sigmoid activation. Subscript $i$ indicates the patient label, $j$ corresponds to the time point, and *k* denotes the feature class. Input data for the LSTM was masked and padded to handle variable sequence lengths. **B** The network was trained M times using longitudinal data from N-1 patients (following a LOOCV procedure), and with a fixed set of hyperparameters. Once the network was trained, we estimated the average probability of risk of PCa progression on AS for the remaining patient

Our data considered the longitudinal patient history of different features as shown in Fig. 2. For the $i-$ th patient, the $k$ input is expressed by the sequence $({x}_{i1\mathrm{k}}, {x}_{i2\mathrm{k}}, . . . ,{x}_{ij\mathrm{k}}, . . ., {x}_{iTik}$), where$k = 1, 2, . . . , K$. These measurements are collected at the time points ${t}_{j}, j=1, 2, . . . , {T}_{i}$ for every input$k$.

Here, we evaluated multivariate longitudinal observations, separated into three different inputs corresponding to a specific feature type (namely, T2WI- and ADC-derived radiomics, and PSA density). The index $k$ described above labels each group of features T2WI, ADC, and PSAd. In turn, each of these groups represents a multivariate input composed of ${d}_{k}$ feature columns, for each $k.$

In this paper, the probability of prostate cancer is estimated from a parallel long short-term memory (LSTM) architecture [22–25]. The architecture is described by the following set of equations:

$${h}_{ijk}={o}_{ijk}\odot \mathrm{tanh}({c}_{ijk})$$

where

$$\begin{array}{c}{c}_{ijk}={f}_{ijk}\odot { c}_{ij-1k}+ {i}_{ijk}\odot \widetilde{{ c}_{ijk}}\\ \widetilde{{c}_{ijk}}=\mathrm{tanh}\left({h}_{ij-1k}{U}_{c}+{x}_{ijk}{W}_{c}+{b}_{c} \right)\\ {i}_{ijk}= \sigma \left({h}_{ij-1k}{U}_{f}+{x}_{ijk}{W}_{i}+{b}_{i} \right)\\ {f}_{ijk}= \sigma \left({h}_{ij-1k}{U}_{f}+{x}_{ijk}{W}_{f}+{b}_{f} \right)\\ {o}_{ijk}= \sigma ({h}_{ij-1k}{U}_{o}+{x}_{ijk}{W}_{o}+{b}_{o} )\end{array}$$

Here, ${i}_{ijk}, {f}_{ijk}$ and ${o}_{ijk}$ refer to the input, forget, and output gates, respectively. $\widetilde{{c}_{ijk}}$ is the candidate cell state, ${c}_{ijk}$ is the current cell state, and ${h}_{ijk}$ is the hidden state. The weight matrices W and U were adjusted during learning along with the biases b. Both W and U had dimensions ${d}_{k}\mathrm{ x} {H}_{k}$ and ${H}_{k}\mathrm{ x} {H}_{k}$, respectively. Here ${d}_{k}$ indicates the number of feature columns of each multivariate input $k$, and ${H}_{k}$ is the number of hidden units; biases have dimension $1$ x ${H}_{k}$; $\odot$ denotes the point-wise multiplication.

For the patient$i$, time-step$j$, and input$k$, we calculated the sequence of hidden states$\left({h}_{i1k, }{h}_{i2k, }\dots , {h}_{ijk }, \dots , {h}_{i{T}_{i}k}\right)$. The last element in the sequence ${h}_{i{T}_{i}k}$ was extracted and then concatenated with other hidden states from the remaining $K-1$ LSTMs to have a single vector,${h}_{i}$, accounting for the whole multivariate time series. That is,

$${h}_{i} = [{h}_{i{T}_{i}1, } {h}_{i{T}_{i}2}, \dots , {h}_{i{T}_{i}K}]$$

Finally, a dropout was added during training followed by a regular densely connected neural network layer of 1 unit with sigmoid activation,

$$\widehat{{o}_{i} }= \sigma ({W}_{l} \widetilde{{h}_{i}}+ {b}_{l})$$

where $\sigma \left(x\right)=1/ (1+\mathrm{exp}-x) \widetilde{{h}_{i}})$, denotes the last hidden state after dropout with a set rate, ${W}_{l}$ is a weight vector of size $\left({H}_{1}+ {H}_{2} + \cdot \cdot \cdot + {H}_{K}\right) \times 1$, and ${b}_{l}$ is a scalar bias. $\widehat{{o}_{i}}$ is the estimated probability to be compared with the target outcome (0 for controls and 1 for cases) using binary cross entropy.

In this study, we had $K= 3$ associated with T2WI-derived radiomic features, ADC-derived radiomic features, and PSA density. Each of these had ${d}_{k }$column features 17, 27, and 1, respectively. Each input was passed to its associated LSTM cell, from where we estimated the probability of cancer.

For the training stage, we used batch gradient descent with dynamic learning rates updated through Adam Optimiser [22]. Since neural networks are sensitive to feature scaling, each feature in the data was standardised appropriately during cross-validation by centring each feature value around its mean and then dividing the difference by its standard deviation. This ensured that training converged faster [26]. This scaling transformation was then kept and applied to subsequent validation samples to estimate the probability of PCa progression.

The kernel weights matrix for the recurrent state was initialised by a random orthogonal matrix, while for the inputs we used a kernel weights matrix initialised using Glorot’s scheme. Bias vectors were initialised at zero [27]. Finally, to handle variable sequence lengths, masking and post-padding were applied to the inputs of the network [28–30].

As a preliminary stage, we performed hyperparameter tuning using random sampling with Leave-One-Out Cross-Validation (LOOCV). LOOCV is a special case of cross-validation where the number of folds equals the number of instances in the dataset. Thus, the learning algorithm is applied once for each instance, using all other instances as a training set and using the selected instance as a single-item test set, which is particularly suitable for small datasets where avoiding overfitting is critical [31]. The performance was determined by the mean of binary cross entropy obtained from the cross-validation procedure. The empirical loss was estimated in each case using different combinations of the number of hidden neurons, dropout rate, number of epochs, and learning rates. From this set of simulations, we selected an optimal set of hyperparameters, which we used to report our results: 130 epochs, learning rate of 0.002, dropout 0.2, 16 units for each separate group of features (PSA density, T2WI radiomics, ADC radiomics).

To estimate the repeatability of the estimations provided by our models, the network was trained multiple times using this set of hyperparameters (100 iterations for each of the patients in the cohort following the LOOCV procedure) from which we obtained a distribution of outputs. Then, we computed the mean probability and variance associated with the estimation. LSTM architectures were implemented in Python 3.8.8 using TensorFlow version 2.7.0 and Keras version 2.7.0.

### Statistical and data analysis

Data normality was assessed using the D’Agostino-Pearson test (threshold *p* ≥ 0.05). Intergroup comparison of patient clinicodemographic characteristics was performed using the Mann–Whitney *U* test and Fisher’s exact test as appropriate. DR, TSR, and PRECISE performance against targeted biopsy results (ground truth) were assessed using standard measures of discrimination, with radiomic models validated using a LOOCV scheme to avoid overfitting given the limited sample size compared to the number of radiomic features. In this study, a true positive result was recorded when any of the above models made a correct prediction of tumour progression that was confirmed histopathologically; a false positive result was noted when a model predicted progression in a patient with histopathologically stable disease. In turn, a true negative result was recorded when a model correctly identified stable disease in a patient with no histopathological tumour progression; a false negative result was recorded when a model falsely predicted stable disease in a patient demonstrating histopathological disease progression. Statistical analysis was performed in R version 3.5.1 (R Foundation for Statistical Computing) using the “pROC” and “reportROC” packages as detailed in Supplementary Information.

## Results

### Study population

**Fig. 3:**
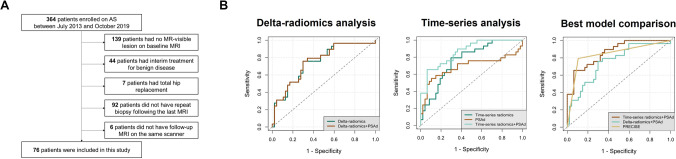
Study flowchart (**A**). ROC curves for standalone and combined radiomic models (**B**) developed as a result of delta-radiomics and time series analyses, along with a summary graph presenting ROC curves for the best-performing models

Of 364 PCa patients enrolled on AS in our centre, 76 were included in this study, with the study flowchart presented in Fig. 3A. Baseline clinicopathological characteristics of the study cohort are listed in Table 1, the final cohort including 28 progressors and 48 non-progressors. Each patient included in this study had only one MR-visible lesion at baseline, and no patient developed new lesions in the follow-up. While baseline gland volume was significantly higher in non-progressors (*p* = 0.004; Table 1), baseline PSAd was significantly increased in progressors (*p* = 0.003; Table 1), further supporting its inclusion in the predictive modelling.

**Table 1:** Baseline clinicopathological characteristics of the study cohort. The data for age, PSA, gland volume, and PSAd are presented as median (interquartile range). *AS* active surveillance, *ISUP* International Society of Urological Pathology, *PSA* prostate-specific antigen, *PSAd* prostate-specific antigen density, *PZ* peripheral zone, *TZ* transition zone

Variable	Total cohort (*n* = 76)	Progressors (*n* = 28)	Non-progressors (*n* = 48)	*p*, progressors vs non-progressors
Age, years	66 (61–69)	66 (59–69)	66 (61–69)	0.79
PSA, ng/mL	5.0 (3.6–7.5)	5.7 (4.0–8.2)	4.5 (3.3–7.3)	0.17
Gland volume, mL	44.8 (36.0–72.0)	39.0 (26.0–47.8)	55.0 (39.3–80.8)	0.004
PSAd	0.10 (0.07–0.17)	0.13 (0.08–0.28)	0.09 (0.06–0.12)	0.003
AS follow-up, mo	42 (31–60)	39 (32–47)	44 (30–68)	0.09
Biopsy ISUP grade 1, *n*	58 (77%)	21 (28%)	37 (49%)	> 0.99
Biopsy ISUP grade 2, *n*	18 (23%)	7 (9%)	11 (14%)	
Target lesion in PZ, *n*	59 (78%)	21 (28%)	38 (50%)	0.80
Target lesion in TZ, *n*	17 (22%)	7 (9%)	10 (13%)	

### Predictive modelling: delta-radiomics, time series radiomics, and PRECISE

Following LOOCV, standalone DR and TSR predictive models achieved AUCs of 0.75 [0.64–0.86] and 0.77 [0.66–0.87], respectively (*p* = 0.714; Fig. 3B), which was similar to the AUC of PRECISE at 0.84 [0.76–0.93] (*p* = 0.108 and 0.199, respectively; Supplementary Fig. 2). The addition of delta-PSAd (0.61 [0.47–0.75]; Supplementary Fig. 3) to DR did not result in a better performance of the combined model (0.75 [0.64–0.87]) compared to the standalone DR model (*p* = 0.503; Fig. 3B). Conversely, the addition of time series PSAd (0.69 [0.55–0.83]; Supplementary Fig. 3) to TSR significantly increased its performance to 0.86 [0.78–0.94] compared to the standalone TSR model (*p* = 0.015; Fig. 3B). As a result, the AUC of the combined TSR + PSAd model was significantly higher compared to that of the DR + PSAd model (*p* = 0.048; Fig. 3B). In addition, the AUCs of both DR + PSAd and TSR + PSAd models were also similar to that of PRECISE (*p* = 0.121 and 0.710, respectively; Fig. 3B). Numerically, TSR + PSAd had the highest specificity, PPV, and AUC of 0.94, 0.86, and 0.86, while PRECISE had the highest NPV at 0.88, with 95% CI for each metric reported in Table 2.

**Table 2:** Summary performance characteristics of the best-performing MRI-derived radiomic models and PRECISE scores for predicting prostate cancer progression on AS. Individual performance characteristics are presented along with their 95% confidence intervals. Values with the best performance across the three approaches are highlighted in bold. *AUC* area under the receiver operating characteristic curve, *DR* delta-radiomics, *NPV* negative predictive value, *PPV* positive predictive value, *PRECISE* Prostate Cancer Radiological Estimation of Change in Sequential Evaluation, *TSR* time series radiomics

Model	Sensitivity	Specificity	PPV	NPV	AUC
DR + PSAd	0.76 (0.60–0.91)	0.70 (0.57–0.83)	0.61 (0.45–0.77)	0.83 (0.71–0.94)	0.75 (0.64–0.87)
TSR + PSAd	0.66 (0.48–0.83)	**0.94** **(0.87–1)**	**0.86** **(0.72–1)**	0.82 (0.71–0.92)	**0.86** **(0.78–0.94)**
PRECISE	**0.79** **(0.65–0.94)**	0.89 (0.81–0.98)	0.82 (0.68–0.96)	**0.88** **(0.78–0.97)**	0.84 (0.76–0.93)

## Discussion

We developed an RNN-based framework for time series analysis of MRI-derived radiomic features and PSAd for the prediction of PCa histopathological progression on AS. The standalone TSR model showed similar performance to both expert-derived PRECISE and conventional DR, which is expected in a dataset where the final time point was linked to the ground truth assessment. This may have artificially increased the delta in progressors, leading to an overoptimistic performance of DR. However, in the real-life AS scenarios, the “final time point” used in DR cannot be known in advance, particularly if a decision to biopsy is based on visible tumour radiological progression that may or may not occur at a given time point. Hence, TSR provides a more viable and unbiased framework capable of tracking real-time personalised longitudinal changes in tumour imaging features at all time points throughout AS. Importantly, the improved performance of TSR with the addition of time series PSAd, which was not seen when delta-PSAd was added to DR, further demonstrates the added value of utilising all available serial clinical data. As a result, the AUC of the most holistic model combining TSR and time series PSAd was the closest to that of an expert-derived PRECISE assessment, and it was also considerably higher than previously reported serial MRI performance pooled across different centres [7]. Finally, some radiomic features that passed the robustness analysis including *Maximum 2D Diameter Row* and *Surface Area* directly correspond to previously identified tumour measurements that are known to differ between progressors and non-progressors [32, 33]. These findings suggest that TSR with or without the addition of time series PSAd may offer a quantitative and interpretable solution to standardising serial MRI assessment in AS, with potential to increase radiologists’ confidence and improve performance across centres and healthcare systems by achieving the expert-derived PRECISE standard.

Limitations of this study include its small sample size, which was dictated by the stringent inclusion criteria related to MRI acquisition, ground truth assessment based on repeat targeted biopsy results, and presence of MRI-visible disease. In future work, we will aim to further develop the TSR framework by including studies performed on different vendors using different study protocols, as well as performing external validation alongside whole-gland radiomic feature extraction to include patients with no visible MRI lesions. Importantly, whole-gland segmentation will also be inclusive of patients developing new lesions over the course of AS, thereby overcoming an important limitation of the present approach. These steps will require even more rigorous pre-processing, calibration, feature selection, and validation steps to ensure that the resulting predictive models are generalisable and robust to factors unrelated to the true tumour progression.

In conclusion, we have developed the first predictive model analysing the combination of radiomic and clinical features in a time series fashion, which outperformed the conventional DR approach and showed comparable performance to expert radiologists. The proposed TSR concept could be applied to any clinical scenario involving serial imaging, ranging from continuous monitoring to evaluating treatment response, thus establishing a novel field in AI-driven radiology research.

## Supplementary Information

Below is the link to the electronic supplementary material.

Supplementary file1 (DOCX 392 KB)

## Data Availability

Access to the de-identified data can be provided upon a reasonable request to the corresponding author and is subject to appropriate regulatory approvals and data transfer agreements.

## Abbreviations

ADC: Apparent diffusion coefficient
AS: Active surveillance
AUC: Area under the receiver operating characteristic curve
DR: Delta-radiomics
ISUP: International Society of Urological Pathology
LOOCV: Leave-one-out cross-validation
LSTM: Long short-term memory
MRI: Magnetic resonance imaging
NPV: Negative predictive value
PCa: Prostate cancer
PPV: Positive predictive value
PSA: Prostate-specific antigen
PSAd: Prostate-specific antigen density
PZ: Peripheral zone
RNN: Recurrent neural network
T2WI: T2-weighted imaging
TSR: Time series radiomics
TZ: Transition zone

## Declarations

### Guarantor

The scientific guarantor of this publication is Prof Tristan Barrett.

### Statistics and biometry

Six of the authors have significant statistical expertise (LR, LA, ZL, TN, AZ, OB).

### Study subjects or cohorts overlap

Some study subjects or cohorts have been previously reported in: 1. Sushentsev N, Rundo L, Blyuss O, et al (2021) Comparative performance of MRI-derived PRECISE scores and delta-radiomics models for the prediction of prostate cancer progression in patients on active surveillance. Eur Radiol 2021 1–10. https://doi.org/10.1007/S00330-021-08151-X 2. Sushentsev N, Rundo L, Blyuss O, et al (2021) MRI-derived radiomics model for baseline prediction of prostate cancer progression on active surveillance. Sci Rep 11:12917. https://doi.org/10.1038/s41598-021-92341-6

### Methodology

- Retrospective
- Observational
- Performed at one institution
