# Supplementary material for: Time series radiomics for the prediction of prostate cancer progression in patients on active surveillance
Source: Eur Radiol. 2023 Feb 7;33(6):3792–800. doi: 10.1007/s00330-023-09438-x (PMC10182165; doi:10.1007/s00330-023-09438-x)
Supplement: Supplementary file 1 — Supplementary file1 (DOCX 392 KB) [file 330_2023_9438_MOESM1_ESM.docx]

**Time-series Radiomics for the Prediction of Prostate Cancer Progression in Patients on Active Surveillance**

**Supplementary Information**

**Supplementary Methods**

**Dataset and study population**

The exclusion criteria were prior treatment for benign disease or interim treatment for PCa, and the presence of total hip replacement or other pelvic metalwork. Stable disease was recorded in patients who showed neither radiological nor histopathological PCa progression over the course of AS, with all MRIs included in the study reported as PRECISE score ≤3 [1].

**MRI acquisition**

Unless clinically contraindicated, intravenous injection of hyoscine butylbromide (Buscopan, 20 mg/mL; Boehringer, Ingelheim am Rhein, Germany) was administered prior to imaging to reduce peristalsis.

| **Parameter** | **Localiser*** | **Axial T1 FSE*** | **Axial T2 FSE*** | **Sagittal T2 FSE** | **Axial DWI*** | **Axial DWI Focus** | **DCE LAVA** |
| --- | --- | --- | --- | --- | --- | --- | --- |
| TE/TR, ms | 20/200 | 30/789 | 102/3743 | 102/3743 | 85/3775 | 60/4000 | min full/4.3 |
| FOV, cm | 30 | 32 | 18 | 22 | 28 | 24 | 24 |
| Matrix | 256 | 512 | 384 | 288 | 128 | 356 | 192 |
| Slice thickness, mm | 3 | 6 | 3 | 1 | 3 | 3 | 3 |
| Gap, mm | 0 | 2 | 0 | 0 | 0 | 0 | 0 |
| Phase | 128 | 320 | 224 | 224 | 128 | 80 | 192 |
| b-values | - | - | - | - | 100, 750, 1400 | 100, 2000 | - |
| Synthetic b-values | - | - | - | - | 2000, 2500 | 2500 | - |
| Scan time, min | 00:35 | 02:32 | 05:22 | 03:13 | 02:42 | 04:52 | 06:22 |

**Supplementary Table 1.** MRI acquisition parameters. TR = repetition time, TE = echo time, FOV = field of view, DCE = dynamic contrast enhancement, FSE = fast spin echo, DWI = diffusion-weighted imaging, LAVA = liver acquisition with volume acceleration. * Denote sequences used as part of the biparametric protocol.

**Ground truth assessment**

Depending on the clinical recommendation, either transrectal (DynaCAD, InVivo Corp, Orlando, FL, USA) or transperineal (Biopsee, Oncology Systems Limited, Shrewsbury, UK) biopsies were performed by three expert urologists with 9-21 years’ experience using MRI/ultrasound fusion.

**Image segmentation and analysis**

To assess the reliability of image segmentation by the readers, we applied ROI morphological perturbations using the “Scipy.ndimage.morphology” functions (binary_opening and binary_closing) in the SciPy version 1.3.2 multi-dimensional image processing package.

**Image calibration and pre-processing**

No resampling was applied for 3D feature computation to avoid interpolation artifacts, and no re-segmentation was applied due to the preferred use of the number of bins over the bin width for image quantisation, as advised in the Image Biomarker Standardisation Initiative (IBSI) guidelines [3] in case of modalities with non-calibrated voxel values, such as MRI. According to prior studies, the selected number of bins was 128 [4, 5]. Feature robustness was assessed by applying ROI morphological perturbations to eliminate features susceptible to segmentation variation [6] and applying the intraclass correlation coefficient (ICC) threshold of >0.8 to eliminate intercorrelated features. Importantly, Spearman’s correlation analysis showed no relationship between any radiomic features and MRI acquisition parameters (*P* > 0.05 for all with no multiplicity correction applied).

| **First-order** |
| --- |
| 10^th^ Percentile |
| 90^th^ Percentile |
| Energy |
| Entropy |
| Interquartile Range |
| Kurtosis |
| Maximum |
| Mean Absolute Deviation |
| Mean |
| Median |
| Minimum |
| Range |
| Robust Mean Absolute Deviation |
| Root Mean Squared |
| Skewness |
| Total Energy |
| Uniformity |
| Variance |
| **Shape-based (3D)** |
| Mesh Volume |
| Voxel Volume |
| Surface Area |
| Surface Area to Volume ratio |
| Sphericity |
| Maximum 3D diameter |
| Maximum 2D diameter (Slice) |
| Maximum 2D diameter (Column) |
| Maximum 2D diameter (Row) |
| Major Axis Length |
| Minor Axis Length |
| Least Axis Length |
| Elongation |
| Flatness |
| **Gray Level Co-occurrence Matrix (GLCM)** |
| Autocorrelation |
| Cluster Prominence |
| Cluster Shade |
| Cluster Tendency |
| Contrast |
| Correlation |
| Difference Average |
| Difference Entropy |
| Difference Variance |
| ID: Inverse Difference |
| IDM: Inverse Difference Moment |
| IDMN: Inverse Difference Moment Normalized |
| IDN: Inverse Difference Normalized |
| IMC1: Informational Measure of Correlation 1 |
| IMC2: Informational Measure of Correlation 1 |
| Inverse Variance |
| Joint Average |
| Joint Energy |
| Joint Entropy |
| MCC: Maximal Correlation Coefficient |
| Maximum Probability |
| Sum Average |
| Sum Entropy |
| Sum Squares |
| **Gray Level Dependence Matrix (GLDM)** |
| Dependence Entropy |
| Dependence NonUniformity |
| Dependence NonUniformity Normalized |
| Dependence Variance |
| Gray Level NonUniformity |
| Gray Level Variance |
| High Gray Level Emphasis |
| Large Dependence Emphasis |
| Large Dependence High Gray Level Emphasis |
| Large Dependence Low Gray Level Emphasis |
| Low Gray Level Emphasis |
| Small Dependence Emphasis |
| Small Dependence High Gray Level Emphasis |
| Small Dependence Low Gray Level Emphasis |
| **Gray Level Run Length Matrix (GLRLM)** |
| Gray Level NonUniformity |
| Gray Level NonUniformity Normalized |
| Gray Level Variance |
| High Gray Level Run Emphasis |
| Long Run Emphasis |
| Long Run High Gray Level Emphasis |
| Long Run Low Gray Level Emphasis |
| Low Grey Level Run Emphasis |
| Run Entropy |
| Run Length NonUniformity |
| Run Length NonUniformity Normalized |
| Run Percentage |
| Run Variance |
| Short Run Emphasis |
| Short Run High Gray Level Emphasis |
| Short Run Low Gray Level Emphasis |
| **Gray Leven Size Zone Matrix (GLSZM)** |
| Gray Level NonUniformity |
| Gray Level NonUniformity Normalized |
| Gray Level Variance |
| High Gray Level Zone Emphasis |
| Large Area Emphasis |
| Large Area High Gray Level Emphasis |
| Large Area Low Gray Level Emphasis |
| Low Gray Level Zone Emphasis |
| Size Zone NonUniformity |
| Size Zone NonUniformity Normalized |
| Small Area Emphasis |
| Small Area High Gray Level Emphasis |
| Small Area Low Gray Level Emphasis |
| Zone Entropy |
| Zone Percentage |
| Zone Variance |
| **Neighboring Gray-Tone Difference Matrix (NGTDM)** |
| Busyness |
| Coarseness |
| Complexity |
| Contrast |
| Strength |

**Supplementary Table 2.** Radiomic features extracted from tumour ROIs prior to robustness assessment. Individual feature description can be found at <https://pyradiomics.readthedocs.io/en/latest/>

| **T2WI-derived features** | **ADC-derived features** |
| --- | --- |
| ***First-order*** | ***First-order*** |
| 10^th^ Percentile | 10^th^ Percentile |
| 90^th^ Percentile | 90^th^ Percentile |
| Energy | Energy |
| Interquartile Range | Interquartile Range |
| Maximum | Maximum |
| Mean | Mean Absolute Deviation |
| Mean Absolute Deviation | Median |
| Median | Minimum |
| Minimum | Range |
| Robust Mean Absolute Deviation | Robust Mean Absolute Deviation |
| Root Mean Squared | Total Energy |
| Total Energy | Variance |
| Variance | ***GLCM*** |
| ***GLCM*** | IMC1 |
| Correlation | Joint Entropy |
| IMC1 | *GLDM* |
| ***GLSZM*** | Dependence NonUniformity |
| Size Zone NonUniformity | ***GLSZM*** |
| **Shape** | Size Zone NonUniformity |
| Maximum 2D Diameter Row | ***NGTDM*** |
|  | Strength |
|  | ***Shape*** |
|  | Flatness |
|  | Least Axis Length |
|  | Maximum 2D Diameter Row |
|  | Minor Axis Length |
|  | Surface Area |
|  | Voxel Volume |

**Supplementary Table 3.** Radiomic features included in the predictive modelling following robustness analysis.

**Prostate-specific antigen predictive modelling**

At each time point, PSAd was calculated as the corresponding PSA value divided over the MRI-derived prostate volume measured using the semi-automated DynaCAD software (DynaCAD, InVivo Corp, Orlando, FL, USA) using the scan closest to the date of a PSA measurement. The area under the receiver-operator curve (AUC) for predicting PCa progression on AS was higher for time-series PSAd compared to time-series PSA (0.69 [95% CI: 0.55-0.83] vs 0.57 [0.43-0.70]; *P* = 0.056; **Supplementary Fig. 1**), which led us to add the former parameter to TSR predictive modelling.


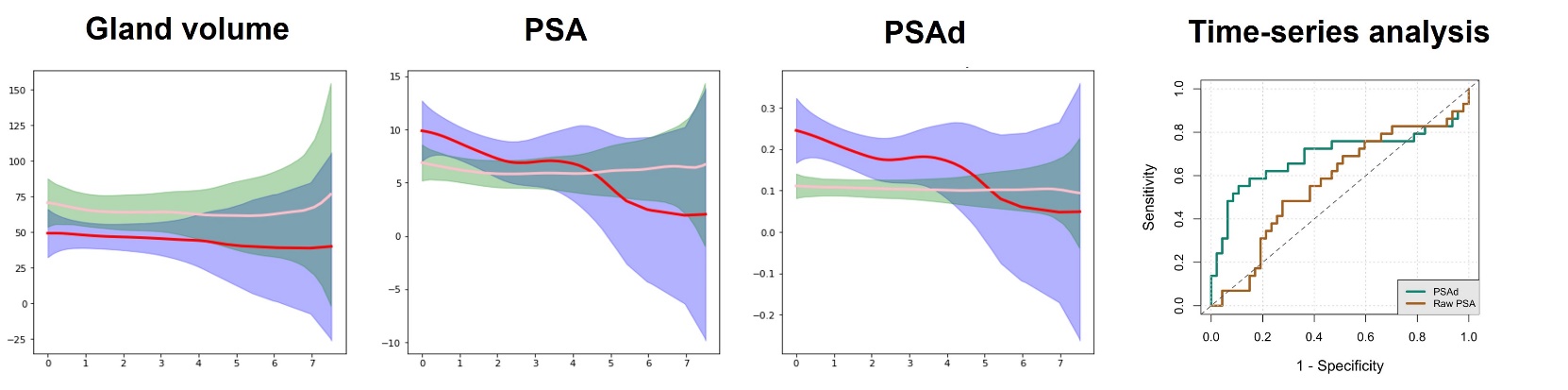


**Supplementary Figure 1.** Time-series measurements of gland volume, PSA, and PSAd in the study cohort. Locally weighted scatterplot smoothing (LOWESS) curves demonstrating time-series changes in the gland volumes, PSA, and PSAd in progressors (red lines) and non-progressors (yellow lines), with x-axes denoting years to progression or end of follow-up and y-axes denoting corresponding measures. A ROC-curve for time-series PSA and PSAd predicting histopathological progression on AS is also presented.

**Statistical and data analysis**

In parenclitic networks, a support vector machine (SVM) with radial basis functions was trained for each pair of features using the “e1071” R package with default settings. For each sample, a network was then built wherein vertices corresponded to features and the edge weight was the disease progression probability as predicted by the SVM classifier. The mean of the vertices degrees was calculated, and a generalised linear model (GLM) classification was then constructed using the “stats” R package with default settings. LSTM architectures were implemented in Python 3.8.8 using the TensorFlow version 2.7.0 and Keras version 2.7.0.

The performance of DR and TSR predictive models was assessed with measures of discrimination using LOOCV and compared with the PRECISE-based model. Specificity and sensitivity were derived using nonparametric stratified resampling with the percentile method (2,000 bootstrap replicates)[13]. In addition, areas under the ROC curve (AUCs) were calculated for each model, alongside 95% confidence intervals using DeLong’s asymptomatically exact method to evaluate the uncertainty of each AUC [14]. AUC values were compared between the models using DeLong’s test for correlated/paired AUCs [14]. Positive and negative predictive values (PPV and NPV, respectively) and their 95% confidence intervals were computed using the standard approach [15].

**Supplementary Results**


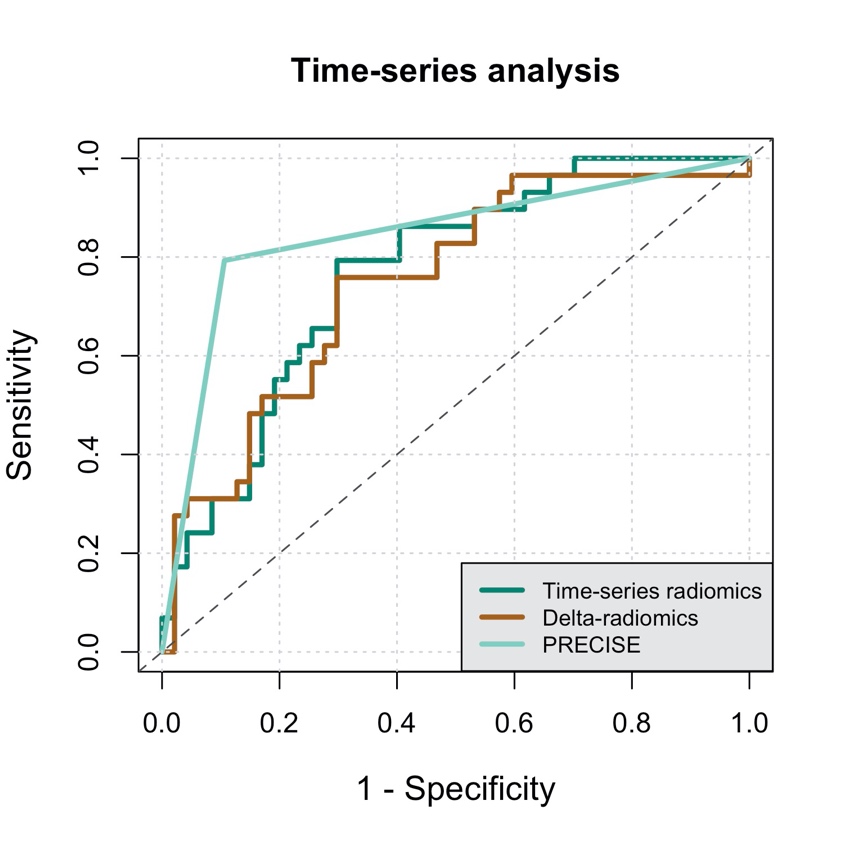


**Supplementary Figure 2.** ROC curves for standalone delta-radiomics (DR), time-series radiomics (TSR), and PRECISE. As described in the main text, none of the resulting AUCs were significantly different from each other.


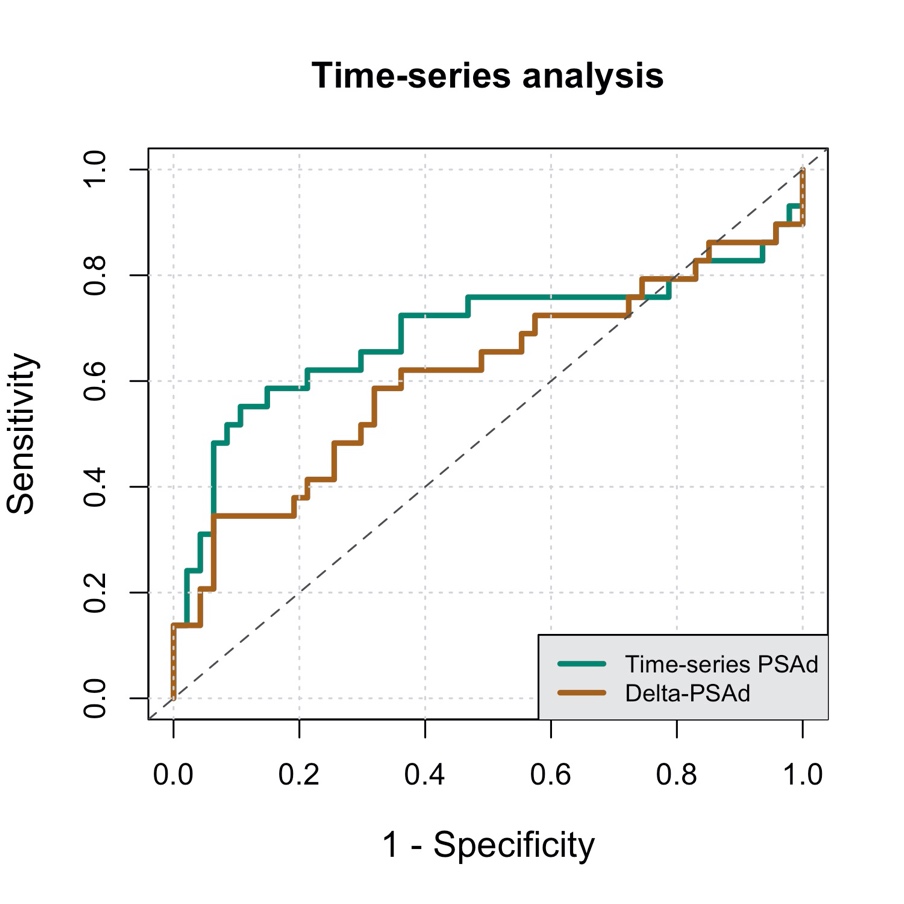


**Supplementary Figure 3.** ROC curves for delta- and time-series PSAd. No significant difference was noted between the AUCs of the two parameters, *P* = 0.445.

**Supplementary references:**

1. Moore CM, Giganti F, Albertsen P, et al (2017) Reporting Magnetic Resonance Imaging in Men on Active Surveillance for Prostate Cancer: The PRECISE Recommendations—A Report of a European School of Oncology Task Force. Eur Urol 71:648–655. https://doi.org/10.1016/j.eururo.2016.06.011

2. Giganti F, Pecoraro M, Stavrinides V, et al (2020) Interobserver reproducibility of the PRECISE scoring system for prostate MRI on active surveillance: results from a two-centre pilot study. Eur Radiol 30:2082–2090. https://doi.org/10.1007/s00330-019-06557-2

3. Zwanenburg A, Vallières M, Abdalah MA, et al (2020) The image biomarker standardization initiative: Standardized quantitative radiomics for high-throughput image-based phenotyping. Radiology 295:328–338. https://doi.org/10.1148/radiol.2020191145

4. Sushentsev N, Rundo L, Blyuss O, et al (2021) MRI-derived radiomics model for baseline prediction of prostate cancer progression on active surveillance. Sci Rep 11:12917. https://doi.org/10.1038/s41598-021-92341-6

5. Sushentsev N, Rundo L, Blyuss O, et al (2021) Comparative performance of MRI-derived PRECISE scores and delta-radiomics models for the prediction of prostate cancer progression in patients on active surveillance. Eur Radiol 2021 1–10. https://doi.org/10.1007/S00330-021-08151-X

6. Zwanenburg A, Leger S, Agolli L, et al (2019) Assessing robustness of radiomic features by image perturbation. Sci Rep 9:1–10. https://doi.org/10.1038/s41598-018-36938-4

7. Chagas P, Souza L, Pontes I, et al (2022) Uncertainty-aware membranous nephropathy classification: A Monte-Carlo dropout approach to detect how certain is the model. https://doi.org/101080/2168116320222029573. https://doi.org/10.1080/21681163.2022.2029573

8. Levasseur LP, Hezaveh YD, Wechsler RH (2017) Uncertainties in Parameters Estimated with Neural Networks: Application to Strong Gravitational Lensing. Astrophys J Lett 850:L7. https://doi.org/10.3847/2041-8213/AA9704

9. Singhal N, Soni S, Bonthu S, et al (2022) A deep learning system for prostate cancer diagnosis and grading in whole slide images of core needle biopsies. Sci Reports 2022 121 12:1–11. https://doi.org/10.1038/s41598-022-07217-0

10. Wickstrøm K, Kampffmeyer M, Jenssen R (2020) Uncertainty and interpretability in convolutional neural networks for semantic segmentation of colorectal polyps. Med Image Anal 60:101619. https://doi.org/10.1016/J.MEDIA.2019.101619

11. Ghoshal B, Tucker A, Sanghera B, Lup Wong W (2021) Estimating uncertainty in deep learning for reporting confidence to clinicians in medical image segmentation and diseases detection. Comput Intell 37:701–734. https://doi.org/10.1111/COIN.12411

12. Saxe AM, McClelland JL, Ganguli S (2013) Exact solutions to the nonlinear dynamics of learning in deep linear neural networks. 2nd Int Conf Learn Represent ICLR 2014 - Conf Track Proc. https://doi.org/10.48550/arxiv.1312.6120

13. Carpenter J, Bithell J (2000) Bootstrap confidence intervals: When, which, what? A practical guide for medical statisticians. Stat Med 19:1141–1164. https://doi.org/10.1002/(SICI)1097-0258(20000515)19:9<1141::AID-SIM479>3.0.CO;2-F

14. DeLong ER, DeLong DM, Clarke-Pearson DL (1988) Comparing the Areas under Two or More Correlated Receiver Operating Characteristic Curves: A Nonparametric Approach. Biometrics 44:. https://doi.org/10.2307/2531595

15. Altman DG, Bland j. M (1994) Statistics Notes: Diagnostic tests 2: Predictive values. BMJ 309:102. https://doi.org/10.1136/bmj.309.6947.102
